# Plasma-Driven Carbon Mobilization Creates a Transient Near-Surface Carbonaceous Interphase for Selective CO_2_ Conversion into CO

**DOI:** 10.1021/acs.jpclett.6c02597

**Published:** 2026-09-21

**Authors:** Julia Moszczynska, Xinying Liu, Marek Wiśniewski

**Affiliations:** † Department of Materials Chemistry, Adsorption and Catalysis, Faculty of Chemistry, Nicolaus Copernicus University in Toruń, Gagarina 7, 87-100 Toruń, Poland; ‡ Institute of Advanced Studies, Nicolaus Copernicus University in Toruń, Wileńska 4, 87-100 Toruń, Poland; § Institute for Catalysis and Energy Solutions, University of South Africa, Christiaan de Wet and Pioneer, Florida 1709, Roodepoort, Gauteng, South Africa; ∥ Institute of Future Technology, 12624 Zhejiang University of Technology, Hangzhou 310014, China

## Abstract

Carbon beds enhance plasma-assisted CO_2_ conversion into resources, but are commonly treated as stationary reactant surfaces. Here, we show that nonthermal plasma (NTP) continuously mobilizes and reconstructs carbon, generating a transient near-surface carbonaceous interphase. In this study, we test these properties of carbon beds as enabling selective CO production during CO_2_ decomposition under low-temperature plasma conditions. Graphene oxide/chitosan-derived carbons, amorphous carbon, and graphite were examined under CO_2_ and Ar using gas-phase FTIR, time-resolved SEM, and Raman spectroscopy. GO@CHT-650 displayed the highest CO_2_ conversion into CO as well as oxygen-scavenging selectivity. Carbonaceous nanodeposits formed under NTP, developed continuously with exposure time, remained confined to the C-surface, and exhibited morphologies and dimensions dependent on the parent carbon structure. Raman analysis showed that the reconstructed phase remained predominantly disordered. These observations link plasma-driven carbon mobility with reactor performance and reveal a catalyst–reactant duality: individual carbon species are consumed stoichiometrically, whereas the dynamically reconstructed interphase persists as the reaction medium. Efficient plasma-assisted CO_2_ conversion into CO, therefore, requires a transient near-surface carbonaceous interphase created and sustained by the plasma itself.

## Graphical abstract

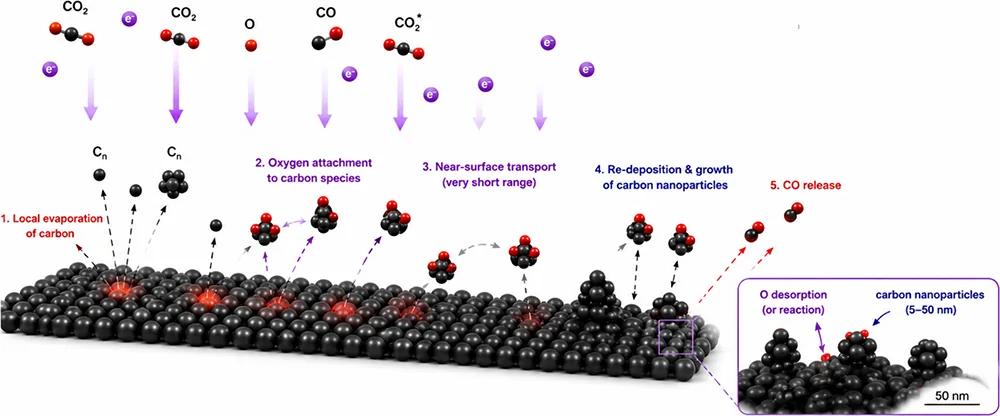

Nonthermal plasma creates a strongly nonequilibrium reaction environment in which energetic electrons activate gas-phase molecules, while ions, radicals, and excited species simultaneously interact with solids exposed to the discharge.^1^ This dual action makes plasma-assisted catalysis mechanistically unusual, because the plasma can modify not only the reaction pathways but also the physical identity of the active material.

This behavior is particularly relevant to CO_2_ treatment, where electron-impact and vibrational excitation can promote molecular dissociation without heating the entire gas to the temperatures required for equilibrium thermolysis.^1^ However, the primary dissociation event represents only part of the overall process. Oxygen atoms and molecules formed together with CO promote back-reactions both within the discharge and in the afterglow, making the final CO_2_ conversion into resources strongly dependent on the fate of the released oxygen.^2^ Carbon surfaces are known to bind and reduce oxygen-containing species through chemisorption and the formation of surface oxygen complexes.^3–5^ Molecular O_2_ can even undergo dissociative chemisorption on carbon at room temperature.^5^ Consistent with this surface chemistry, carbon beds can intercept plasma-generated O/O_2_ species, thereby suppressing their recombination with CO, while unconverted CO_2_ generates additional CO through the reverse Boudouard reaction: CO_2_ + C → 2CO.

Accordingly, coupling plasma reactors with biochar or activated carbon beds has been shown to enhance CO_2_ conversion, enrich the product stream in CO, and decrease the amount of oxygen leaving the reactor.^6–10^

In the prevailing mechanistic description, carbon is treated as a stationary solid that performs three closely related functions: a surface for CO_2_ adsorption and activation, an oxygen scavenger, and a stoichiometric reductant consumed through carbon gasification.^6–10^ This description is supported by kinetic models based on surface oxygen complexes and by the need to replenish carbon during prolonged operation.^7–10^ In our previous in situ FTIR study, carbon materials were likewise shown to act simultaneously as adsorbents and oxygen scavengers during plasma-assisted CO_2_ treatment.^8^ Excited adsorbed CO_2_ species and surface C–O and C=O functionalities were identified as reaction intermediates. These observations revealed the surface chemistry of the process but were interpreted within the conventional picture of reactions occurring on a structurally stationary carbon surface. Whether carbon exposed to plasma actually remains such a surface was not addressed.

A distinctly different picture emerges from plasma–carbon interaction studies. Bombardment of graphite by plasma species can release carbon atoms and clusters, followed by their nucleation, growth, transport, and redeposition as carbonaceous nanoparticles.^11,12^ Substantial erosion and local redeposition have also been observed during direct plasma exposure of graphite, with both surface-grown structures and particles formed within the plasma phase.^13^ These mobile carbon species are conventionally described as plasma dust, erosion products, or unwanted deposits rather than as components of an active reaction phase. Conversely, heterogeneous catalysis provides examples in which reaction-generated carbonaceous deposits become catalytically active instead of merely deactivating the original catalyst.^14^ Nevertheless, these concepts have not been connected in plasma-assisted CO_2_ conversion into resources: carbon-bed studies assume a stationary reactive solid, whereas plasma-material studies recognize mobile carbon but do not assign it a role in the chemical transformation of CO_2_.

This study investigated the critical functions of a carbon bed in the plasma CO_2_ reduction process. We propose that plasma exposure transforms the nominally static carbon surface into a transient near-surface carbonaceous interphase. We use “*interphase*” intentionally to denote a region of finite thickness and dynamically evolving composition, rather than an ideal two-dimensional carbon surface. This interphase is continuously generated through local carbon mobilization, interacts with activated CO_2_ and oxygen-containing species, and undergoes short-range redeposition within the carbon bed. Individual carbon species can therefore participate stoichiometrically in CO formation, while the reconstructed carbonaceous phase persists at the macroscopic scale and controls both reaction rate and product stoichiometry. Carbon consequently exhibits a catalyst–reactant duality: it is consumed at the atomic level but functions as a dynamically regenerated reaction medium at the macroscopic level.

We examine this hypothesis using graphene oxide/chitosan-derived carbons denoted GO@CHT-X, where X corresponds to the carbonization temperature (650 or 750 °C), together with amorphous carbon and graphite, under CO_2_ and Ar nonthermal plasma conditions. Gas-phase analysis is combined with time-resolved postexposure electron microscopy and Raman spectroscopy to correlate CO_2_ conversion and CO/CO_2_ product stoichiometry with carbon mobilization and redeposition. We show that carbon deposits formed even under Ar plasma; their dimensions systematically depended on the structural ordering of the parent material, and their accumulation evolved with plasma exposure time while remaining confined to the carbon bed. These observations support a mechanism in which plasma-assisted CO_2_ conversion into resources is governed not solely by reactions *on* carbon but by the plasma-driven transformation of carbon into a transient, spatially confined, and continuously reconstructed reactive interphase.

The functional consequences of introducing the carbon materials into the plasma zone are shown in Figure 1. In the reactor without a carbon bed, CO_2_ conversion remained within approximately 37–45% over the investigated voltage range. Both tested carbon samples increased the conversion, although their effects were substantially different. GO@CHT-750 yielded CO_2_ conversions of approximately 55–67%, whereas GO@CHT-650 maintained values above 85%, reaching approximately 98% at 8 kV. Thus, both carbon materials enhanced the level of CO_2_ conversion, and the magnitude of this enhancement depended strongly on the structural state of the material.

**Figure 1:**
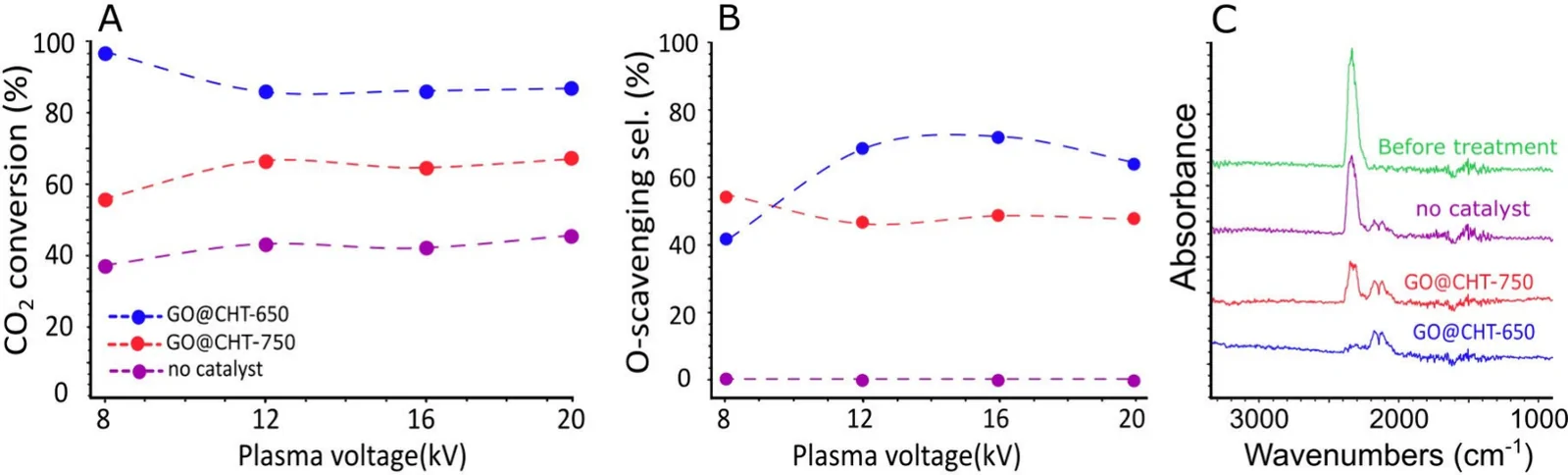
CO_2_ conversion into CO (A) oxygen-scavenging selectivity, S­(O) (B), and sample FTIR spectra (C), during nonthermal plasma treatment in the absence of a carbon bed and in the presence of GO@CHT-650 or GO@CHT-750.

The product stoichiometry requires a more precise descriptor than conventional CO selectivity because CO was the only detected carbon-containing gaseous product. For direct plasma splitting,

1 $${\mathrm{CO}}_{2}\to \mathrm{CO}+1/2O_{2}$$

The molar CO gain equals the CO_2_ loss. By contrast, complete transfer of the released oxygen to carbon gives the limiting stoichiometry,

2 $${\mathrm{CO}}_{2}+C\to 2\mathrm{CO}$$

for which the CO gain is twice the CO_2_ loss. We can define this stoichiometry as the ratio of CO gain to CO_2_ loss (R­(CO)).

3 $$R\left(\mathrm{CO}\right)=\left|\frac{\Delta n\left(\mathrm{CO}\right)}{\Delta n\left({\mathrm{CO}}_{2}\right)}\right|$$

and the apparent oxygen-scavenging selectivity as

4 S(O)=100[R(CO)−1]

Accordingly, (S­(O) = 0%) corresponds to (R­(CO) = 1), whereas (S­(O) = 100%) corresponds to (R­(CO) = 2).

For the reactor without a carbon bed, S­(O) was zero by definition (Figure 1B), consistent with the stoichiometry of direct CO_2_ splitting. The introduction of either carbon material shifted the product balance toward R­(CO) > 1. GO@CHT-750 exhibited an oxygen-scavenging selectivity of approximately 49–56%, with only a weak dependence on the voltage. For GO@CHT-650, S­(O) increased from approximately 43% at 8 kV to 68–75% between 12 and 20 kV. The simultaneous enhancement of CO_2_ conversion and shift in the CO/CO_2_ product stoichiometry demonstrate that the carbon bed influences both CO_2_ activation and the fate of the oxygen-containing species generated by the plasma. This behavior is consistent with previous reports showing that carbon beds promote plasma-assisted CO_2_ conversion and intercept plasma-generated oxygen species.^6–10^

Worth noting that all comparisons in Figure 1 were performed in the same reactor using identical bed geometry, filling fraction, gas flow, pressure, electrode arrangement, and applied voltage. Because the electrical and dielectric properties of the carbon beds may influence the current, field distribution, and deposited power, a fixed applied voltage does not necessarily correspond to an identical energy input to the discharge. Figure 1 should therefore be interpreted as a comparison of the overall bed-dependent responses of the coupled plasma-carbon systems under matched external operating conditions, rather than as a power-normalized comparison of energy efficiency or intrinsic catalytic activity. Possible material-induced changes in discharge behavior are consequently included in the observed system response, together with the chemical and structural properties of the carbon bed.

Because GO@CHT-650 and GO@CHT-750 were derived from the same precursor system but carbonized at different temperatures, their contrasting performance points to an important role of the carbonization-induced structural state in governing plasma-carbon interactions. The morphology and structural organization of both materials were therefore examined before and after plasma exposure by using SEM and Raman spectroscopy.

SEM imaging revealed pronounced plasma-induced reconstruction of both carbon surfaces following CO_2_-NTP treatment (Figure 2). Before exposure, both materials exhibited folded and wrinkled morphologies. After plasma treatment, both materials were covered by nanoscale carbonaceous structures that were absent before exposure (Figure 2A′, B′). Their morphology, however, differed markedly between the two materials. On GO@CHT-650, the deposits formed a dense and relatively uniform nanoparticulate coverage distributed along the original surface. In contrast, GO@CHT-750 developed spatially heterogeneous, three-dimensional agglomerates that extended above the underlying carbon surface. Plasma exposure, therefore, did not produce a generic carbon coating; instead, the carbonization-induced structural state of the parent material influenced the spatial organization and growth mode of the redeposited carbonaceous phase. The formation of these distinct deposits demonstrates that the material exposed to the discharge cannot be regarded as a permanently stationary surface.

**Figure 2:**
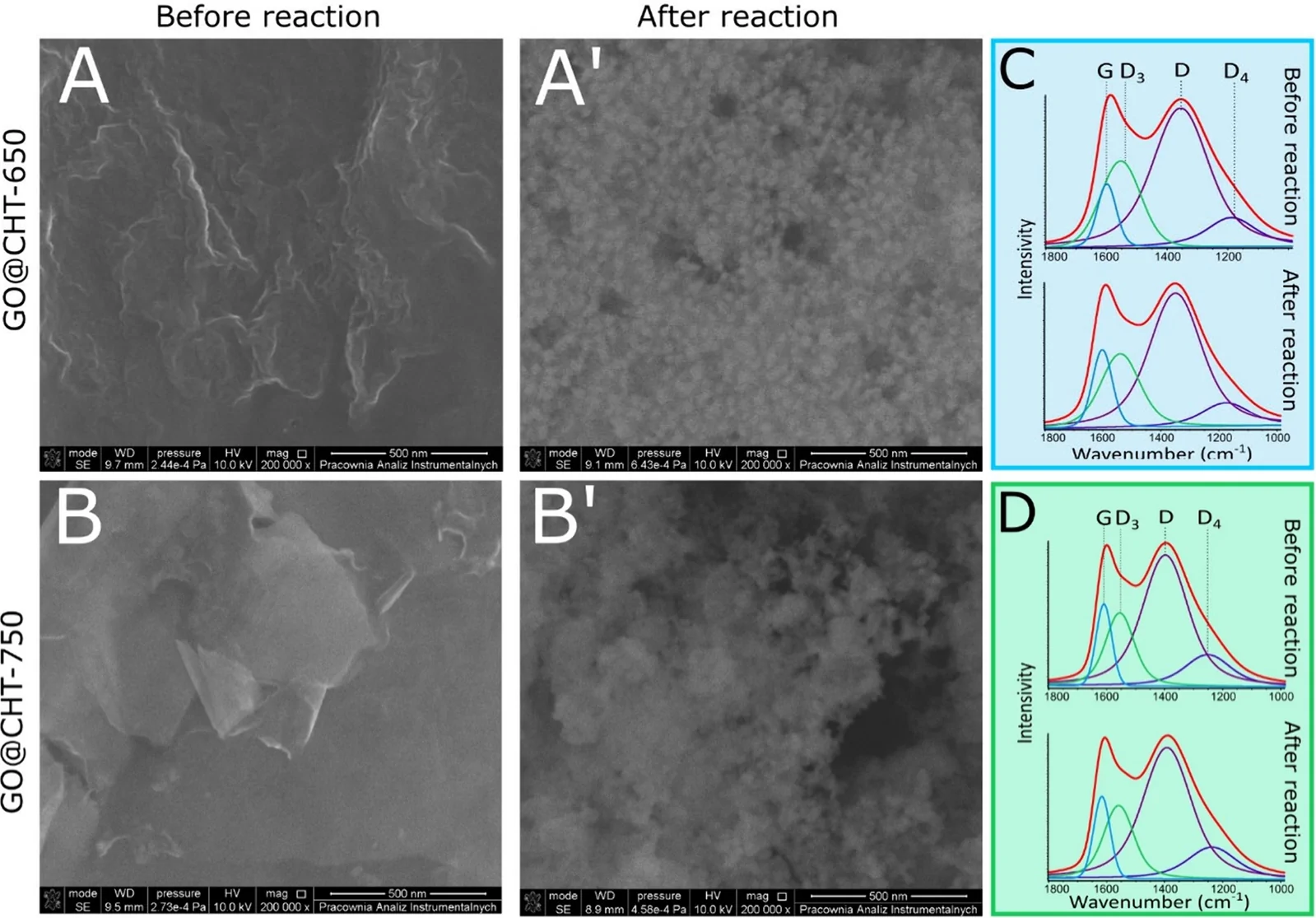
Plasma-induced reconstruction of GO@CHT-derived carbon surfaces. SEM images of GO@CHT-650 (A, A′) and GO@CHT-750 (B, B′) before and after CO_2_-NTP treatment, respectively. Panels C and D show the corresponding Raman spectra deconvoluted into G, D, D_3_, and D_4_ contributions before and after 3 h of CO_2_-NTP exposure.

Raman spectroscopy provided complementary information on the structural character of this reconstruction (Figure 2C and D). Before and after NTP treatment, both materials exhibited broad D- and G-band envelopes containing the D_3_ and D_4_ contributions characteristic of structurally disordered carbon.^17^ Despite the markedly different deposit morphologies observed by SEM, the after-reaction spectra retained a similar overall component structure and showed no evolution toward a more-ordered graphitic signature. The plasma-generated layers can therefore be described as predominantly disordered carbonaceous phases, whose growth mode and spatial organization depend on the parent material.

The SEM and Raman results demonstrate that the carbon bed does not behave as a permanently stationary surface during the plasma-assisted CO_2_ treatment. Instead, plasma exposure drives carbon mobilization and redeposition, producing a reconstructed near-surface carbonaceous interphase whose morphology is governed by the initial carbon architecture.

Deposits formed during the CO_2_ treatment could, in principle, originate either from the carbon material or from reactions involving gas-phase CO_2_. To distinguish between these possibilities, plasma exposure was repeated under Ar, where no carbon-containing gas reactant was present. Plasma-induced sputtering, erosion, cluster release, transport, and redeposition are well-established for graphite and other carbon targets.^11–13,15,16^ Depending on the plasma conditions and material structure, the mobilized species can include carbon atoms, clusters, and nanoscale fragments. The present experiment does not distinguish unambiguously among sputtering, fragmentation, ion-assisted erosion, and thermally assisted release; these processes are therefore collectively described as plasma-driven carbon mobilization.

Four materials spanning different degrees of structural ordering were investigated under identical Ar-NTP conditions: GO@CHT-650, GO@CHT-750, cellulose-derived amorphous carbon (C-600), and graphite. Carbonaceous nanoparticles appeared on every material after 3 h of exposure, demonstrating that their formation does not require CO_2_ and that the deposited carbon cannot originate solely from the gas-phase reactant (Figure 3). The particle size distributions nevertheless depended strongly on the parent material. GO@CHT-650 produced the broadest distribution, extending from approximately 20 to 60 nm. The particles formed on GO@CHT-750 were predominantly below approximately 30 nm, whereas the structures observed on graphite were concentrated mainly below approximately 20 nm. C-600 carbon exhibited an intermediate distribution.

**Figure 3:**
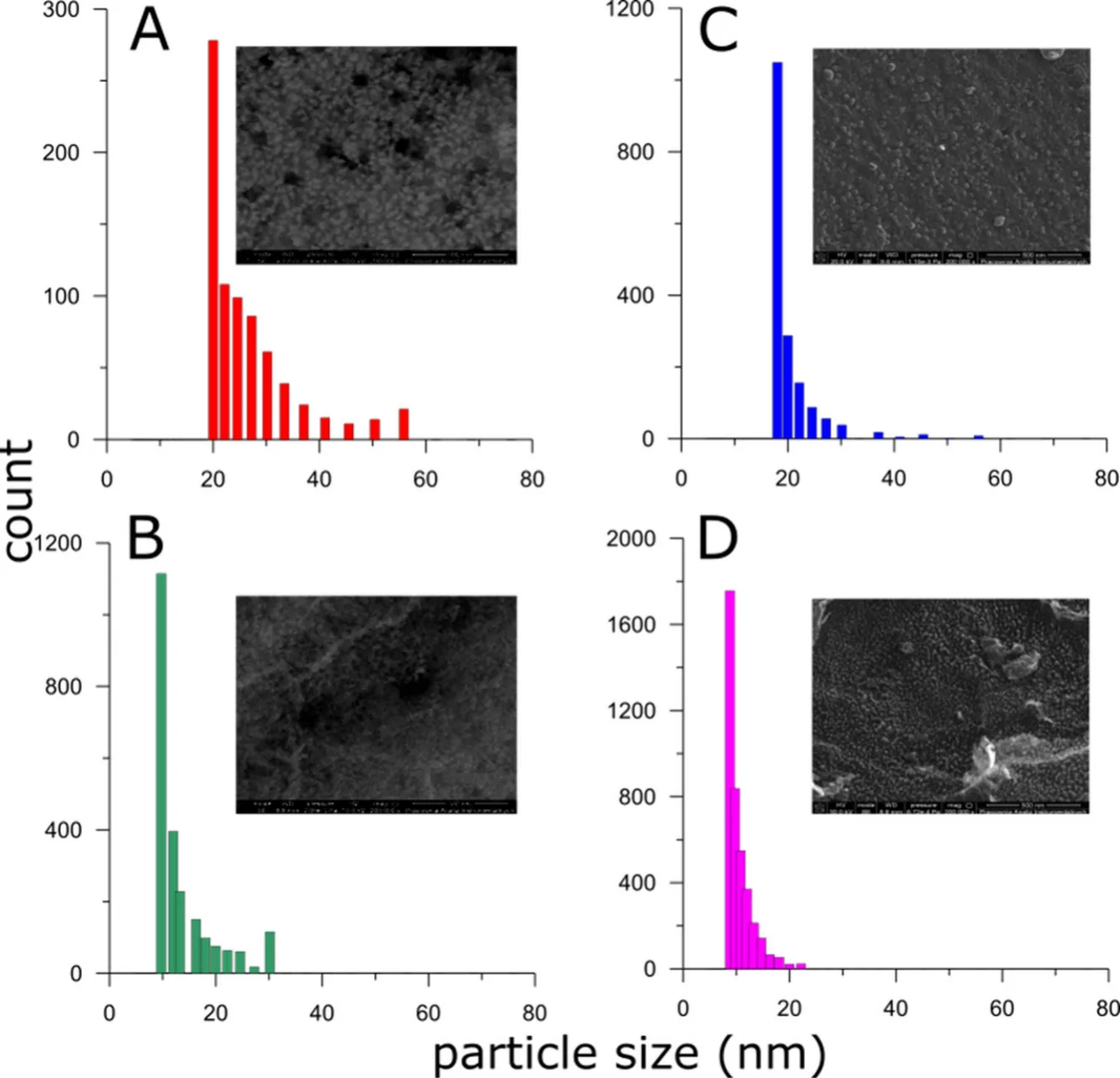
Particle-size distributions of carbonaceous deposits formed after 3 h of Ar-NTP exposure on GO@CHT-650 (A), GO@CHT-750 (B), C-600 (C), and graphite (D) at 10 mbar and 20 kV. The insets show representative SEM images used for particle-size analysis.

Because the histograms contain different total particle counts, their comparison is based primarily on particle size ranges and distribution shapes rather than absolute particle abundance.

The qualitative decrease in the deposit size with increasing structural ordering suggests that the organization of the parent carbon affects either the release of carbon species, their local concentration, or their subsequent aggregation. Fewer ordered carbons contain a larger population of edge sites, defects, strained domains, and weakly connected carbon fragments that may facilitate plasma-driven mobilization. A larger local flux or longer residence of such species would promote collisions, aggregations, and the formation of larger deposits. Conversely, the more-ordered structure of graphite may limit the population of readily mobilized carbon species, resulting in smaller redeposited structures.

The spatial localization of the deposits provides further support for the near-surface character of the reconstructed carbonaceous phases. After plasma treatment, the glass reactor walls upstream and downstream of the carbon bed remained visually clean (Figure S1), whereas carbonaceous deposits developed on the materials within the bed (Figure 2). Together with the material-dependent particle size distributions, this spatial confinement indicates that carbon mobilization, aggregation, and redeposition occur predominantly over short distances in the immediate vicinity of the parent material. The resulting reactive carbonaceous phase can therefore be described as a localized near-surface interphase rather than as a deposit transported through the reactor.

The temporal evolution of the deposits was subsequently examined under an Ar plasma (Figure 4). After 5 min, only isolated nanoscale structures were visible on the carbon surface. After 1 h, their surface density increased substantially, and after 3 h, numerous particles and interconnected agglomerates covered large regions of the samples. Because these experiments were conducted under a constant Ar flow without a carbon-containing gas-phase reactant, the accumulated structures cannot be described as conventional reaction products. Instead, the sequence represents a cumulative record of continuous carbon mobilization, short-range transport, aggregation, and redeposition.

**Figure 4:**
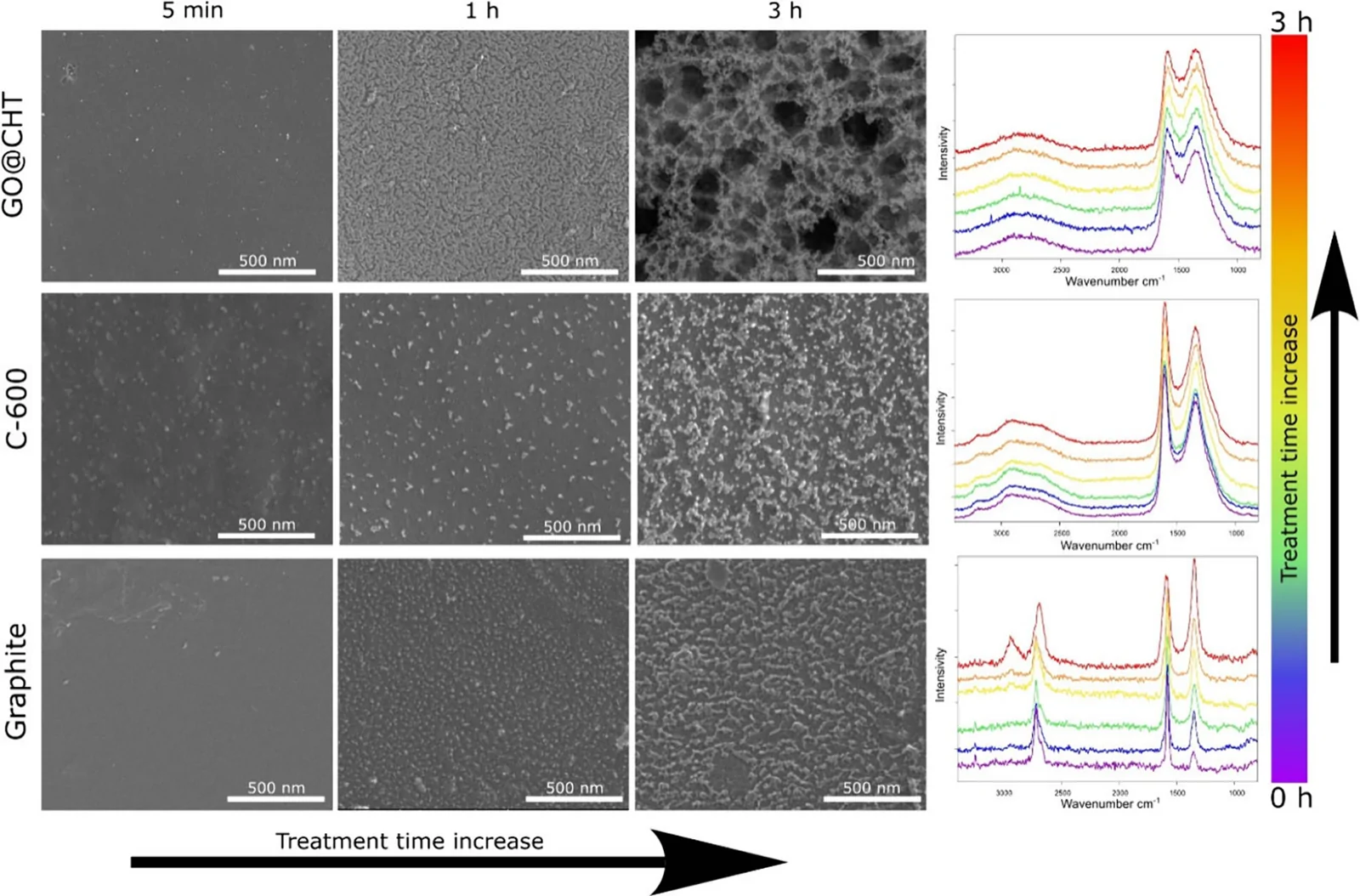
Time-dependent evolution of carbonaceous deposits on GO@CHT, C-600, and graphite during Ar-NTP exposure at 10 mbar and 20 kV. SEM images were collected after 5 min, 1 h, and 3 h of treatment. The corresponding Raman spectra show the evolution of the D- and G-band regions as a function of plasma exposure time.

Raman spectroscopy provided complementary evidence of the progressive formation of a disordered carbonaceous phase. For the initially disordered carbons, the ID/IG ratio remained approximately constant during Ar-NTP exposure, indicating that the newly formed surface structures retained a disordered sp2-rich character similar to that of the parent materials. By contrast, the evolution was particularly evident for graphite, for which ID/IG increased from approximately 0.3 before treatment to values above 1 after prolonged plasma exposure (Figure S2). This pronounced growth of the defect-activated D band demonstrates the progressive development of a less ordered, amorphous-like carbonaceous phase on the initially graphitic material.^17^ The Raman evolution therefore closely follows the time-dependent accumulation observed by SEM and supports continuous reconstruction of the carbon surface through the formation and redeposition of structurally disordered carbon species.

The time series is important, because it shows that the deposits are not produced by a single initial erosion event. Instead, carbon mobilization and redeposition continue throughout the plasma exposure. The carbon surface, therefore, evolves dynamically: it acts simultaneously as the source of mobile carbon species, the region in which these species interact and aggregate, and the substrate onto which they redeposit.

The combined results reveal a coherent relationship among the structural organization of carbon, its plasma-driven reconstruction, and reactor performance. The GO@CHT-650 sample, which produced the largest redeposited structures and the broadest particle size distribution under Ar plasma, also exhibited the highest CO_2_ conversion and the strongest shift toward the oxygen-scavenging stoichiometric limit at 12–20 kV. In comparison, the more structurally ordered GO@CHT-750 formed smaller deposits and showed a correspondingly lower conversion and a weaker shift in product stoichiometry. These parallel trends indicate that carbon’s ability to generate and reorganize mobile carbonaceous species is an important material property linking its initial structure to its function under plasma conditions. The formation of deposits under Ar, their systematic dependence on the parent carbon structure, their continuous development with exposure time, and their spatial confinement to the bed collectively support a dynamic picture in which the carbon surface is continuously mobilized and reconstructed into a reactive near-surface interphase.

A mechanistic model consistent with these observations is presented in Figure 5. Under Ar plasma, bombardment by energetic plasma species mobilizes carbon atoms, clusters, or nanoscale fragments from the parent material. These species remain close to the carbon surface, where they can collide, aggregate, and redeposit (Figure 5A):

5 $$C\left(s\right)\overset{\mathrm{plasma‐driven mobilization}}{\to }C_{n}^{\mathrm{mobile}}$$

6 $$C_{n}^{\mathrm{mobile}}+C_{m}^{\mathrm{mobile}}\overset{\mathrm{aggegation}}{\to }C_{n+m}^{\mathrm{mobile}}$$

7 $$C_{n}\overset{\mathrm{redeposition}}{\to }C\left(s\right)$$

**Figure 5:**
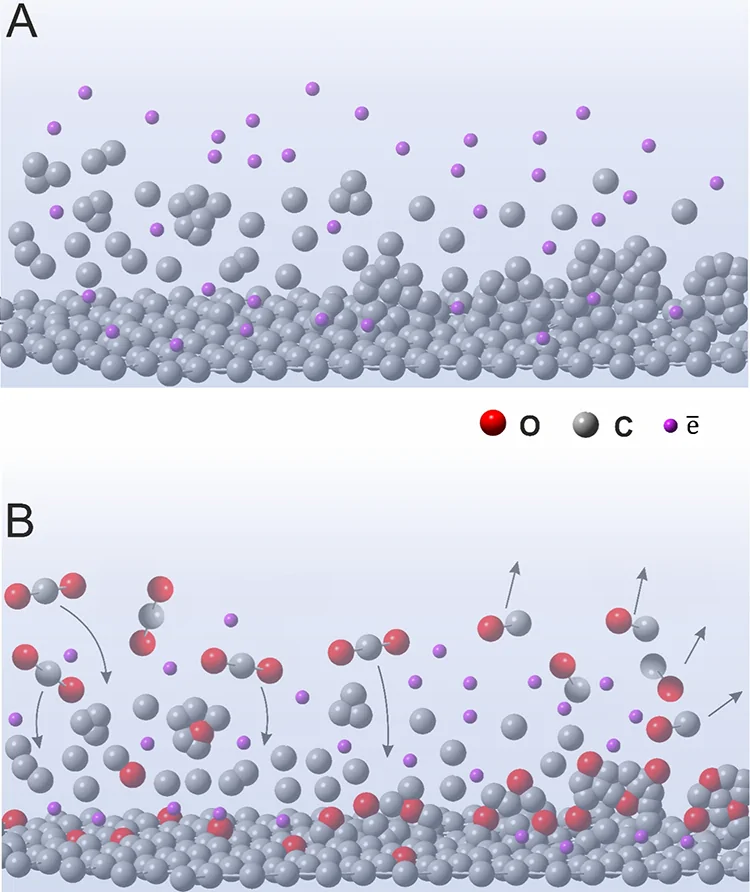
Proposed model of the transient near-surface carbonaceous interphase. (A) Plasma-driven mobilization, aggregation, and short-range redeposition of carbon species under Ar. (B) Interaction of the dynamically reconstructed carbonaceous interphase with activated CO_2_ and oxygen-containing species. Individual carbon species are consumed during CO formation, whereas the interphase persists through continuous mobilization and redeposition.

Under CO_2_ plasma, the same mobile carbon population coexists with electronically or vibrationally activated CO_2_*, CO, O atoms, and other oxygen-containing species. Direct plasma dissociation produces CO together with oxygen:

8 $${\mathrm{CO}}_{2}^{*}\to \mathrm{CO}+O$$

The released oxygen can be intercepted by mobile or surface-associated carbon species:

9 $$O+C_{n}\to C_{n}\left(O\right)$$

Alternatively, activated CO_2_ may react directly with a mobile carbon species through an oxygenated carbon intermediate:

10 $${\mathrm{CO}}_{2}^{*}+C_{n}\to \mathrm{CO}+C_{n}\left(O\right)$$

followed by

11 $$C_{n}\left(O\right)\to \mathrm{CO}+C_{n-1}\left(O\right)$$

The oxygenated Cn­(O) species in this scheme represent a family of transient oxygen-containing carbon structures rather than a single identified molecular intermediate. Their inclusion is consistent with the established formation of surface oxygen complexes on carbons^3–5^ and with our previous in situ FTIR observations of C–O and C=O species during plasma-assisted CO_2_ conversion into resources.^8^ The present SEM and Raman measurements do not directly resolve the composition or lifetime of the mobile intermediates, but they demonstrate the continuous formation and reconstruction of the carbonaceous environment in which such reactions can occur.

At the level of an individual carbon species, the net reaction remains stoichiometric:

12 $${\mathrm{CO}}_{2}+C_{n}\to 2\mathrm{CO}+C_{n-1}$$

Carbon is therefore consumed and cannot be classified as a conventional, mass-conserving catalyst. At the macroscopic level, however, the near-surface population of mobile, aggregated, oxygenated, and redeposited carbon species is continuously reconstructed and controls both the rate of CO_2_ conversion and the CO/CO_2_ product stoichiometry. This establishes a catalyst–reactant duality.

We describe this active region as a transient near-surface carbonaceous interphase rather than a carbon cloud or an ideal surface. The “*near-surface*” reflects the absence of visible long-range deposition outside the carbon bed; “*carbonaceous*” allows for both elemental and oxygenated carbon species; and “*interphase*” denotes a dynamically evolving region of finite thickness containing mobile fragments, aggregates, surface-bound intermediates, and redeposited carbon. This model reconciles the stoichiometric consumption of carbon with its persistent macroscopic influence on reaction rate and product distribution.

In this mechanistic description, “plasma-driven” does not imply an athermal or isothermal surface process. Energy delivered by the nonequilibrium plasma to the conductive carbon bed may be transferred through energetic-species bombardment, local electric-field concentration, microdischarges, and locally generated heat. These phenomena are coupled components of plasma–material interaction rather than mutually exclusive thermal and nonthermal pathways. The present results do not quantify their individual contributions, but this distinction does not affect the observed transition from a stationary carbon surface to a mobilized and continuously reconstructed carbonaceous phase. Moreover, reconstruction under Ar demonstrates that CO_2_ gasification and the reverse Boudouard reaction are not required for this transformation to occur.

In summary, the results presented here demonstrate that a carbon bed does not remain structurally stationary during nonthermal plasma exposure. Plasma-driven mobilization of carbon atoms, clusters, and nanoscale fragments, followed by their aggregation and short-range redeposition, produces a continuously evolving, predominantly disordered carbonaceous phase even under Ar. The size, morphology, and spatial organization of this phase depend on the structural state of the parent carbon. Accordingly, GO@CHT-650, which exhibited the most pronounced redeposition signature, also provided the highest CO_2_ decomposition and the strongest shift toward the oxygen-scavenging stoichiometric limit. These observations identify the transient near-surface carbonaceous interphase as the functional reaction environment that connects carbon structure to reactor performance. Within this interphase, individual carbon species participate stoichiometrically in oxygen consumption and selective CO formation, while the carbonaceous phase is continuously reconstructed through mobilization, aggregation, and redeposition and therefore maintains its macroscopic influence on reaction rate and product stoichiometry, because of catalyst–reactant duality. Rather than representing inactive plasma dust or incidental erosion products, the mobile carbon species constitute an integral component of the CO_2_ conversion pathway. Plasma-assisted CO_2_ conversion into resources therefore proceeds not only on a carbon surface but within a transient carbonaceous interphase created and sustained by the plasma itself.

More broadly, the near-surface interphase replaces the conventional picture of fixed surface active sites with a transient population of mobile atomic- and subnanometric carbon species, opening a pathway toward dynamically generated single-atom and few-atom reactivity under plasma conditions.

## Experimental Methods

Graphene oxide (GO) was synthesized using a modified Hummers method, as described previously.^18,19^ Briefly, graphite (0.15 g) was added to a mixture of concentrated sulfuric acid (32 mL) and concentrated phosphoric acid (8 mL). The suspension was stirred magnetically while potassium permanganate (0.9 g) was added portionwise, and the mixture was then stirred for 24 h. The reaction mixture was poured into distilled water (300 mL) followed by the addition of 30% hydrogen peroxide (5 mL). The resulting suspension was washed to a neutral pH by repeated centrifugation and decantation.

The GO/chitosan composites were prepared as reported previously.^20^ Chitosan (2.0 g) was dissolved in 200 mL of a 2% aqueous acetic acid solution and stirred magnetically until a homogeneous, gel-like solution was obtained. GO was then incorporated into the chitosan matrix at 1 wt % relative to chitosan. The mixtures were vigorously stirred until homogeneous, cast onto flat substrates using the solution-casting method, and dried under controlled conditions to form thin films. After complete solvent removal, the materials were carbonized under a nitrogen atmosphere at either 650 or 750 °C, yielding GO@CHT-650 and GO@CHT-750, respectively.

SEM analysis was performed using a Quanta 3D FEG microscope operated at an accelerating voltage of max 30 kV. Powdered samples were deposited onto carbon tabs attached to aluminum SEM stubs and examined without a conductive coating.

Raman spectra were collected as described previously.^18^ Unpolarized spectra of the carbon samples were recorded over the range 60–4500 cm^–1^ in backscattering geometry using a SENTERRA micro-Raman spectrometer. A 532 nm laser was focused onto the sample surface through a 20× microscope objective. To prevent sample damage, the excitation power was limited to 0.2 mW. A spectral resolution of 4 cm^–1^, a CCD temperature of 223 K, a laser spot diameter of approximately 10 μm, and a total integration time of 100 s (10 × 10 s) were used. The sample position was controlled piezoelectrically in the *XY* plane.

Gas-phase FTIR measurements were performed online by using a gas cell with an optical path length of 0.5 m connected to the reactor flow system and a Mattson Genesis II FTIR spectrophotometer. CO_2_ was treated in a quartz tubular glow-discharge reactor equipped with graphite electrodes with applied voltages of up to 20 kV. The discharge was generated between the electrodes, and the carbon materials were positioned inside the tube between the electrodes. The experimental setup is shown in Figure S1. To exclude the electrode’s influence on the reaction results, a reference measurement was performed with a copper electrode. The results are shown in Figure S3. Additionally, the specific surface areas of graphite and the chitosan material tested as a catalyst were compared (Figure S4). The results clearly exclude the influence of graphite from the electrode on the conversion of CO_2_ to CO and the selectivity of oxygen scavenging. The reactor dimensions and bed arrangement are added in Figure S1. The measured current was 33 mA; the base pressure was 0.01 mbar, and the working pressure of 6.5 mbar was controlled via a damping valve at the inlet of the IR gas cell. The gas flow rate was 20 mL/min.

Blank measurements were performed by replacing the carbon material with a geometrically equivalent bed of chemically inert glass beads positioned in the same reactor region and occupying the same packing volume. This inert-bed control preserved the bed geometry and the generic effects of packing on the gas residence, mixing, and discharge volume.

The concentrations of CO_2_ and CO in the effluent were determined from the integrated absorbances of their respective FTIR bands using previously established calibration curves. CO_2_ was quantified using the band centered at approximately 2343 cm^–1^. The spectrum recorded immediately before plasma ignition was used to determine the initial CO_2_ concentration. The percentage CO_2_ conversion was subsequently calculated from the decrease in CO_2_ concentration during plasma operation. Oxygen-scavenging selectivity was calculated from the ratio of CO gain to CO_2_ loss according to eqs 1–4.

## Supplementary Material



## Notes

Scheme of experimental setup; Additional Raman data; Comparison of CO_2_ conversion and gas-phase CO signals obtained using graphite and copper electrodes; Low-temperature N_2_ adsorption isotherms of the tested catalyst and graphite (PDF)
