# Supplementary material for: Plasma-Driven Carbon Mobilization Creates a Transient Near-Surface Carbonaceous Interphase for Selective CO2 Conversion into CO
Source: J Phys Chem Lett. 2026 Sep 21;17(39):11271–8. doi: 10.1021/acs.jpclett.6c02597 (PMC13637084; doi:10.1021/acs.jpclett.6c02597)
Supplement: Supplementary file 1 [file jz6c02597_si_001.pdf]

Supporting Information

For

Plasma-Driven Carbon Mobilization Creates a Transient Near-Surface Carbonaceous Interphase for Selective CO_2 Conversion into CO

Julia Moszczynska[1,2], Xinying Liu[3,4], Marek Wiśniewski[1,2,*]

1) Department of Materials Chemistry, Adsorption and Catalysis, Faculty of Chemistry, Nicolaus Copernicus University in Toruń, Gagarina 7, 87-100 Toruń, Poland.

2) Institute of Advanced Studies, Nicolaus Copernicus University in Toruń, Wileńska 4, 87-100 Toruń, Poland.

3) Institute for Catalysis and Energy Solutions, University of South Africa, c/o Christiaan de Wet & Pioneer, Florida 1709, Roodepoort, Gauteng, South Africa.

4) Instute of Future Technology, Zhejiang University of Technology, Hangzhou 310014, China.

Corr. Author email: marekw@umk.pl

4 pages
4 Figures

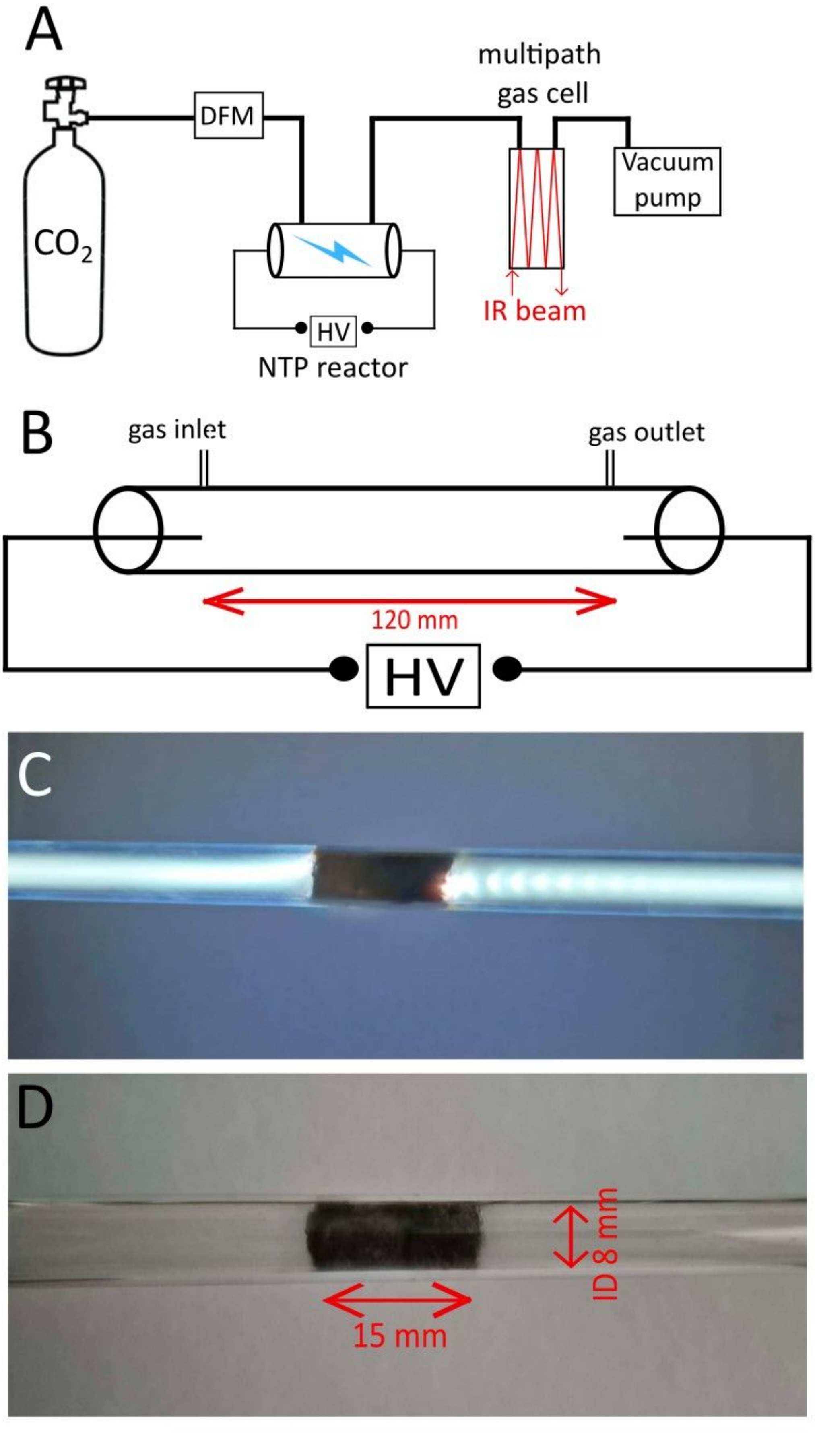

Figure S1. (A) Schematic of the experimental setup; photographs of the reactor (B) during plasma operation and (C) after treatment.

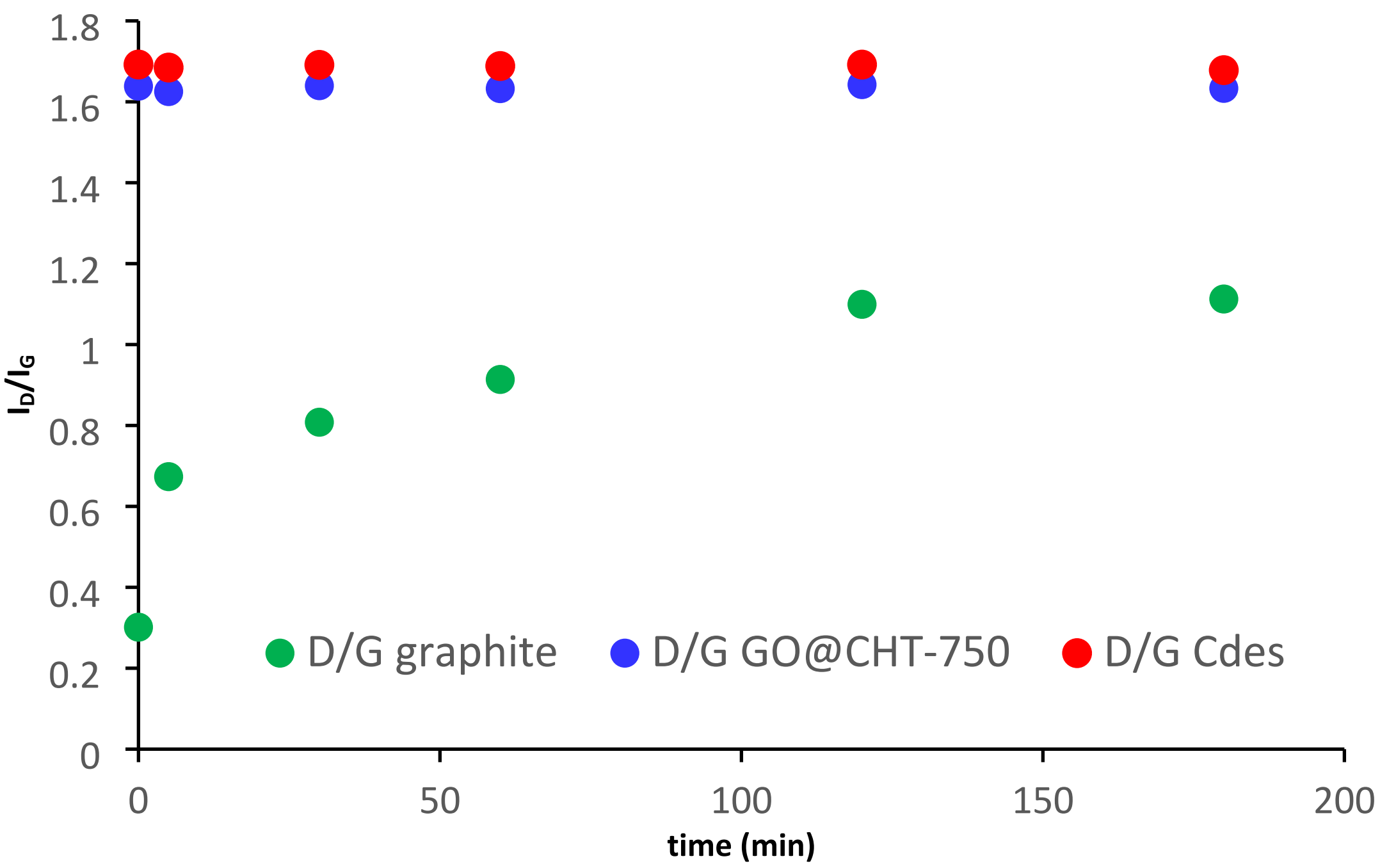

Figure S2. D/G band-intensity ratio for the investigated carbon materials as a function of plasma-exposure time.

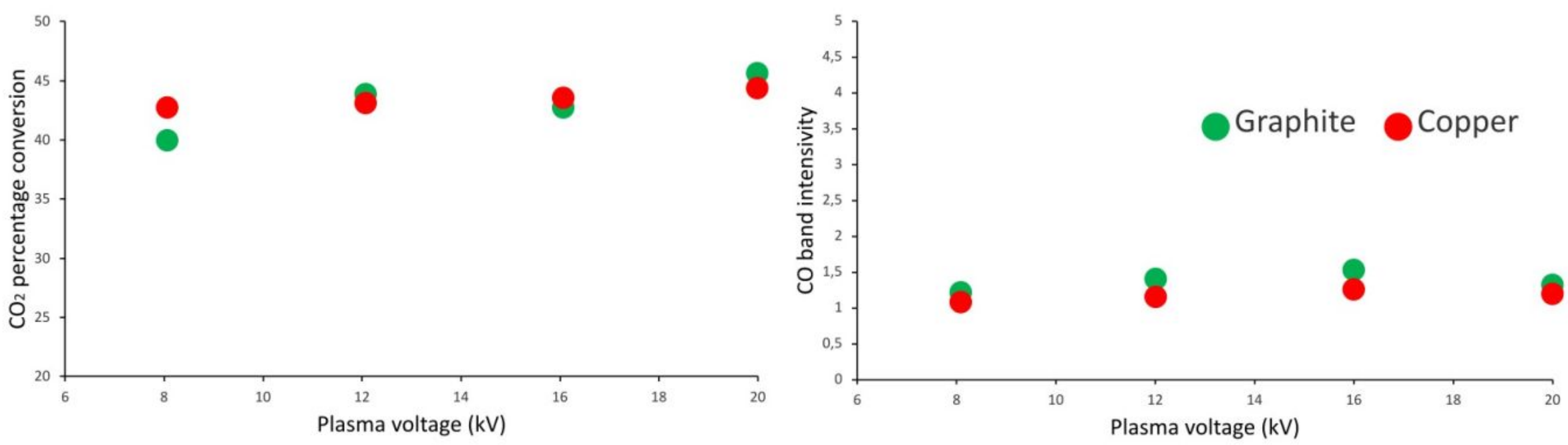

Figure S3. *CO_2 conversion (left) and integrated absorbance of the gas-phase CO band (right) measured during plasma-assisted CO_2 conversion using graphite (green) and copper (red) electrodes as a function of applied voltage. The reactor geometry and other operating parameters were maintained constant; pressure: 6.5 mbar.*

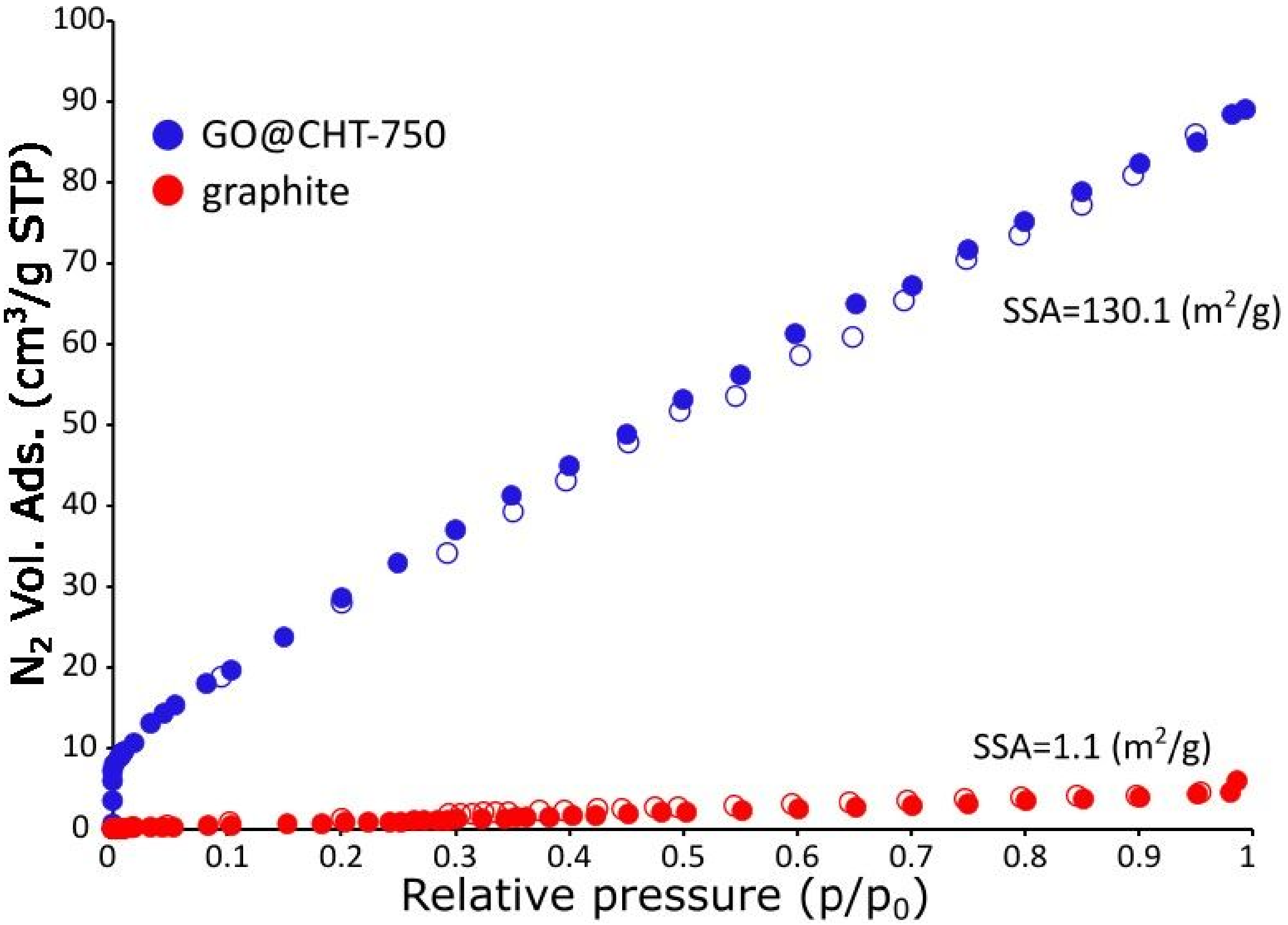

Figure S4. N_2 Low-temperature adsorption isotherms of the tested catalyst and graphite, which was the electrode material.
